# Predicting Power Conversion Efficiency of Organic Photovoltaics: Models and Data Analysis

**DOI:** 10.1021/acsomega.1c02156

**Published:** 2021-09-06

**Authors:** Andreas Eibeck, Daniel Nurkowski, Angiras Menon, Jiaru Bai, Jinkui Wu, Li Zhou, Sebastian Mosbach, Jethro Akroyd, Markus Kraft

**Affiliations:** † CARES, Cambridge Centre for Advanced Research and Education in Singapore, 1 Create Way, CREATE Tower, #05-05, 138602 Singapore; ‡ CMCL Innovations, Sheraton House, Castle Park, Cambridge CB3 0AX, U.K.; § Department of Chemical Engineering and Biotechnology, University of Cambridge, Philippa Fawcett Drive, Cambridge CB3 0AS, U.K.; ∥ School of Chemical Engineering, Sichuan University, Chengdu, Sichuan 610065, China; ⊥ School of Chemical and Biomedical Engineering, Nanyang Technological University, 62 Nanyang Drive, 637459 Singapore

## Abstract

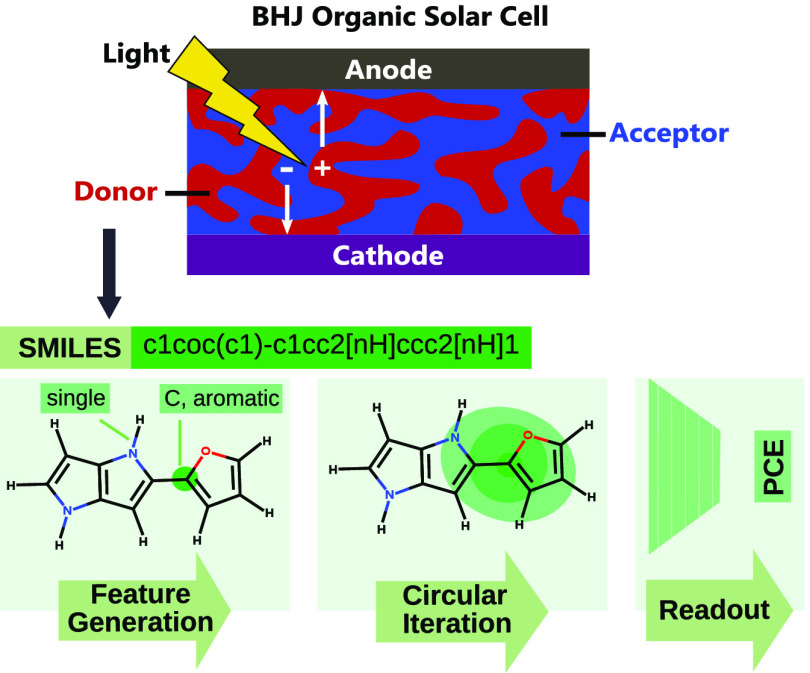

In this paper, the ability of three selected machine learning neural and baseline models in predicting the power conversion efficiency (PCE) of organic photovoltaics (OPVs) using molecular structure information as an input is assessed. The bidirectional long short-term memory (gFSI/BiLSTM), attentive fingerprints (attentive FP), and simple graph neural networks (simple GNN) as well as baseline support vector regression (SVR), random forests (RF), and high-dimensional model representation (HDMR) methods are trained to both the large and computational Harvard clean energy project database (CEPDB) and the much smaller experimental Harvard organic photovoltaic 15 dataset (HOPV15). It was found that the neural-based models generally performed better on the computational dataset with the attentive FP model reaching a state-of-the-art performance with the test set mean squared error of 0.071. The experimental dataset proved much harder to fit, with all of the models exhibiting a rather poor performance. Contrary to the computational dataset, the baseline models were found to perform better than the neural models. To improve the ability of machine learning models to predict PCEs for OPVs, either better computational results that correlate well with experiments or more experimental data at well-controlled conditions are likely required.

## 1 Introduction

With a strong global push toward clean energy generation, more resources are being invested in researching and developing photovoltaic devices. While silicon-based solar cells remain the most prominent in the solar cell market, other materials have also been rapidly gaining interest, such as perovskite-based solar cells^1,2^ that have been seen to achieve promising power conversion efficiencies (PCEs).^3^ However, perovskite-based cells are known to have environmental stability and processing issues.^4^ As a consequence, organic solar cells (OSCs) have been gaining interest, due to their low weight, flexibility, environmental stabilities, and ease of manufacture.^5−7^ Although OSCs often have substantially lower PCEs,^6,8^ recent synthesis efforts and theoretical predictions have suggested that OSCs could achieve conversion efficiencies that make them competitive with silicon and perovskite-based materials,^9−11^ with potential PCEs reaching as high as 20%^9^ or even 30%^11^ in some cases.

As conducting experiments can prove to be challenging both time and resourcewise, computational methods are often employed to enable rapid screening of candidate materials for organic solar cells based on PCE. Frequently, computational estimates of the PCE employ the widely used Scharber equation,^6^ which predicts the PCE of a given organic solar cell architecture from only a few key parameters, all of which can be determined by application of quantum chemical methods such as density functional theory (DFT). However, DFT calculations require substantial computational time that is not conducive to fast screening. Therefore, machine learning (ML) methods are often used to derive quantitative structure–property relationships (QSPR) between the performance of the organic photovoltaic and the underlying properties of the materials, as they can make use of existing computational and experimental data and make predictions at a fraction of the cost.

A wide variety of machine learning algorithms have been applied to predict the performance of organic photovoltaics using different target datasets. The Harvard clean energy project database (CEPDB),^8^ is one such target dataset for ML models that contains computationally determined PCE values for 2.3 million organic photovoltaic candidates. An example of ML methods being applied to the CEPDB is the artificial neural network (ANN) trained by Pyzer-Knapp et al.,^12^ who achieved good prediction accuracy for PCE and other molecular properties. Various deep learning models have also been applied, including the convolutional neural network of Sun et al.,^13^ who classified organic photovoltaic candidates into low-performance (<5% PCE) and high-performance (5–10% PCE) materials, as well as various graph neural network (GNN) approaches that directly predict PCE.^14−16^ Recent approaches have integrated the attention mechanism, originally introduced for recurrent neural networks for machine translation,^17^ to improve the performance by focusing on local substructures that are relevant for the prediction task. For example, Wu et al.^18^ used an attention layer to couple a bidirectional long short-term memory (LSTM),^19^ and multilayer perceptron (MLP) to predict PCE based on sequentialized molecular structure and fragment types. The authors achieved a very high degree of prediction accuracy on the CEPDB and identified functional groups that contribute toward a molecule having higher PCE.

However, it has been noted that computational predictions of PCE of OSCs often do not agree well with experimental measurements, and that machine learning approaches like Gaussian process regression are necessary to improve agreement.^8,20,21^ As a consequence, experimental OPV datasets, such as the Harvard organic photovoltaic dataset (HOPV15),^22^ are often used to train ML methods instead. Examples include the *k*-nearest neighbors (*k*-NN) and Kernel ridge regression (KRR) models used for direct PCE prediction by Padula et al.,^23^ ANN and RF methods used for classification by Nagasawa et al.,^24^ and the five ML models (MLP, deep neural network (DNN), convolutional neural network, RF, and support vector machine (SVM)) trained by Sun et al.^25^ In general, ML models perform worse on experimental data than the CEPDB, with better performing models reaching Pearson correlation coefficients *r* of 0.7. Given this, studies have also tried to include additional DFT-computed molecular descriptors in the ML models to improve performance on experimental datasets. This includes the training of RF, ANN, and gradient boosting regression trees by Sahu et al.,^26^ Sahu and Ma^27^ in conjunction with 13 DFT-derived molecular descriptors and 300 experimental PCEs, as well as the work by Zhao et al.,^28^ who trained SVM, *k*-NN, and KRR models with a variety of different DFT-derived descriptors and 566 experimental PCEs. The performance of these ML models was similar, achieving correlations of *r* = 0.7–0.8, and suggests that including DFT descriptors achieves only a modest improvement, possibly due to many descriptors being implicitly linked to the structure.

Ultimately, the goals of these studies is to train an ML model that can accurately and quickly screen candidate OPV materials to identify potential high-performance candidates for further characterization. While it appears that several different ML approaches can achieve similar levels of performance, a crucial aspect is the choice of training data. Training to computationally determined PCEs has the advantage of large and standardized datasets with controllable and known degrees of freedom,^29^ but these PCEs correlate poorly with experimental measurements, which can undermine their utility. However, training to experimentally characterized PCEs is harder due to the wide variety of experimental conditions, expected experimental errors, larger number of degrees of freedom, and usually smaller amount of available data.

The bulk of literature studies focus primarily on developing more sophisticated machine learning models to achieve better predictions of PCE. However, even though several studies do achieve high predictive capability on computational datasets, less attention is given to the suitability of the datasets themselves and whether these predictions would agree with experimental PCE measurements. In light of these considerations, the aim of this paper is to critically test the ability of machine learning models to predict the PCE of organic photovoltaics based on the SMILES-derived molecular structure information, as well as assess the impact and implications of the choice of training data. To do this, three neural machine learning models are trained: the BiLSTM model used by Wu et al.,^18^ the attentive fingerprints (FP) used by Xiong et al.,^15^ and a simple graph neural network (simple GNN) that serves as an intermediate between these two models in terms of featurization included. Three baseline models: random forests (RF), support vector regression (SVR), and high-dimensional model representation (HDMR) are also trained for comparative purposes. These six models are trained to predict PCE based on descriptions of the molecular structure generated from SMILES strings and fingerprint analysis. To test the impact of training data, the six models are trained to both the large, entirely computational CEPDB, and the small, experimental HOPV15 dataset. Finally, the impact of the training data and choice of the ML model on predicting PCEs that are ultimately useful for guiding organic photovoltaic design is critically assessed based on the training results.

## 2 Data

### 2.1 Harvard Clean Energy Project Dataset

The first major dataset used in this work is the Harvard CEPDB, developed originally by Hachmann et al.^8^ There are two main reasons for utilizing CEPDB in this work. First, it is the most widely used computational dataset in the literature, and has been the target of numerous studies that train various models to achieve prediction of PCE.^12−16,18^ Thus, applying the models in this work to the CEPDB allows confirmation that the performance is comparable to the state of the art that is reported in the literature. Second, using the models on a computational dataset provides a point of juxtaposition when the same models are used on experimental datasets. This should help determine whether models that achieve good prediction on computational data truly give predictions that reflect measured PCE values.

As mentioned previously, the CEPDB only contains computational results for approximately 2.3 million organic solar cell acceptor candidate materials. While the original servers and websites for the CEPDB are no longer in use, the data can be accessed from the following website: https://www.matter.toronto.edu/basic-content-page/data-download. The CEPDB provides substantial data for training machine learning algorithms. The molecular structures in the CEPDB are generated from 26 different building blocks, as detailed in Hachmann et al.^8^ Each species in the CEPDB is assigned an ID, with the stoichiometry and SMILES string also provided to give the basic molecular structural information for the organic solar cell acceptor species. For each species, the PCE, the short-circuit current density *J*_sc_, the open-circuit voltage *V*_oc_, the HOMO energy, the LUMO energy, and the HOMO–LUMO gap are reported as computed by the DFT methods described by Hachmann et al.^8^ The PCE values reported in the CEPDB are computed using the Scharber equation
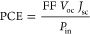
1where FF is the fill factor and *P*_in_ is the input power. Scharber et al.^6^ also developed models for the various parameters in the Scharber equation, which are used to derive the CEPDB data. The fill factor, FF is assumed to be 0.65, and the open-circuit voltage given by the following expression, derived by Scharber et al.^6^
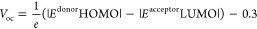
2with *e* as the electron charge, *E*^donor^HOMO being the energy of the highest occupied molecular orbital (HOMO) of the donor material in the cell, *E*^acceptor^LUMO similarly being the energy of the lowest unoccupied molecular orbital (LUMO) of the acceptor material in the cell, and 0.3 being an empirical correction. In the CEPDB, the acceptor is assumed to be PCBM, a class of fullerene derivatives commonly used as electron acceptors in both hybrid perovskite and organic photovoltaic materials. The magnitude of *E*^acceptor^LUMO is assumed to be 4.3 eV, in accordance with experimental measurements.^30^

The two remaining parameters in the Scharber equation, *J*_sc_, and *P*_in_, are both derived from the incident solar photon flux density, which essentially amounts to integrating the air mass 1.5 (AM1.5) spectra.^31^ For this spectra, integrating the spectra across the wavelength gives a *P*_in_ of approximately 1000 W/m^2^. The short-circuit current density is given by
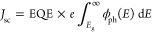
3with the external quantum efficiency EQE set to 0.65 in the Scharber model, *E*_g_ as the band gap of the donor material, and ϕ_ph_ being the incident solar photo flux density as a function of energy *E*.

The Scharber model shows that computing PCE, *V*_oc_, and *J*_sc_ only requires determining the HOMO and LUMO levels of the donor material in the OSC, which is readily derived from the quantum chemical calculations performed by Hachmann et al.^8^

Before implementing and training the various machine learning algorithms to the data in the CEPDB, the data is first preprocessed to understand the characteristics of the species in the CEPDB. First, all of the provided SMILES strings are checked to see if they are valid, in that they successfully encode a sensible molecular structure that can be identified by RDKit. In total, 9821 invalid SMILES were identified and thus these species were removed from consideration for training purposes. Next, the maximum length of the valid SMILES, the maximum number of atoms in a single molecule in the dataset, and the different atom types are identified, as these define the bounds of the structures that the machine learning methods will need to model. For the CEPDB, the maximum length of SMILES and maximum number of atoms in a single molecule are 83 and 53, respectively. There are seven different atoms in the CEPDB, namely C, H, O, N, Si, and Se. However, several atoms can be either aromatic or nonaromatic. This can significantly impact the underlying chemistry and molecular behavior, which is another factor to be taken into account. A final layer of preprocessing is related to the reported PCE values, in which molecules with a PCE value smaller than the defined threshold value of 0.0001 are also removed from consideration, as such species are not going to be useful for OSC applications. This resulted in another 109 425 species being removed from the CEPDB set, resulting in the 2 203 603 remaining species to be used as potential training data.

After the CEPDB is preprocessed, stratified sampling is then applied to derive a set of 25 000 candidate OSC donors for training and testing the machine learning models, as utilizing the full preprocessed CEPDB is computationally prohibitive. A set size of 25 000 species is both computationally affordable and large enough for the training of machine learning models to be tractable, and is in line with previous literature studies.^18^ In this case, the maximum PCE value in the preprocessed CEPDB is 11.13%. As such, the preprocessed data are first assigned to 56 bins of equal widths of 0.2%, with the first bin including species with PCE [0,0.2) and the last bin including species with PCE [11.0,11.2). Once the preprocessed data is divided into the 56 bins, stratified sampling without replacement is then performed with respect to these bins. This is done so that the PCE profile of the selected 25 000 species is close to the PCE profile for the entire preprocessed dataset. Once the 25 000 species are selected, this is then divided into a set of 15 000 species for training the machine learning method, a validation set of 5000 species used to evaluate the ML model fit while tuning hyperparameters, and a test set of 5000 species used to give the final evaluation of the ML model fit.

### 2.2 Harvard Organic Photovoltaic Dataset

The second major dataset used in this work is the HOPV15 dataset, which consists of 350 different experimentally characterized organic solar cell donor structures that have been collated from various studies in the literature by Lopez et al.^22^ The HOPV15 data is accessible as a single file from the original publication.^22^ For each of the 350 species, the HOPV15 dataset contains the SMILES and InChI strings that define the molecular structure, as well as the DOI for the original work that experimentally characterized the species in question. The experimental data in HOPV15 also includes the construction type of the donor species, the architecture of the solar cell, the complement used (i.e., the acceptor material), and any measured photovoltaic characteristics of the organic solar cell. These photovoltaic characteristics include the HOMO and LUMO energies of the donor, the electrochemical and optical gaps of the donor, as well as the PCE, *J*_sc_, *V*_oc_, and fill factor, but not all of these properties are measured for all 350 species in HOPV15.

The HOPV15 dataset contains experimental results from a variety of different experimental conditions, which can be seen in the breakdown of the solar cell characteristics. There are 127 solar cells that made use of molecular constructions for the donor, while 220 used polymeric constructions for the donor species. In terms of the architecture, 270 experiments involved bulk heterojunction solar cells, 13 made use of bilayer solar cells for measurements, and 64 used dye-sensitized-solar cells (DSSCs). The acceptor material used also varies, unlike in CEPDB. In this case 139 experiments made use of PC61BM as an acceptor, 133 used PC71BM, 9 used C60, 64 used TiO_2_, 1 used ICBA, and 1 used PDI. For three species in the HOPV15, no experimental information was provided at all.

Given the variety of experiments used to generate the data in HOPV15, preprocessing and sampling are again applied to the data before any training and testing of machine learning algorithms is performed. As with CEPDB, the SMILES strings are first checked for validity. In this case, all 350 SMILES strings are able to be processed using RDKit. The second preprocessing step removes species that do not have experimental PCEs, which excludes 7 species. This leaves 343 species to be sampled from. It is worth noting that for these species, there is an extra atom type compared to CEPDB, namely, that of fluorine, meaning 8 different atoms are possible. Additionally, while the smallest species in HOPV15 contains just 12 atoms, the largest contains 142 atoms, and the average size of species is roughly 71 atoms, meaning that in general the donor candidates here are much larger than the species in CEPDB. This, in addition with the extra atom type mean that HOPV15 contains substantially different structures for the ML methods to model when compared to CEPDB.

Although it is very affordable to train to the entirety of the HOPV15 data, as this involves just 343 PCE values and SMILES strings to be processed, there is the additional issue of the variability of experiments and solar cell setups used to measure these PCEs. As a consequence, not all of the measured PCEs in HOPV15 are directly comparable as they are in the CEPDB, due to the differences in experimental methodology. Therefore, only PCEs measured using similar experimental conditions were selected to try and get a consistent dataset. Specifically, since the CEPDB PCE values assume a PCBM-type material for the acceptor, PCEs measured with a solar cell that did not use PCBM or similar materials as the acceptor, namely, those that used TiO_2_ were excluded. Similarly, only PCEs measured for solar cells using a bulk heterojunction architecture were chosen to remove this variable from the dataset. This sampling meant that 267 PCEs were selected from the HOPV15 dataset to provide a more consistent set of experimental values to train the ML methods.

**Figure 1:**
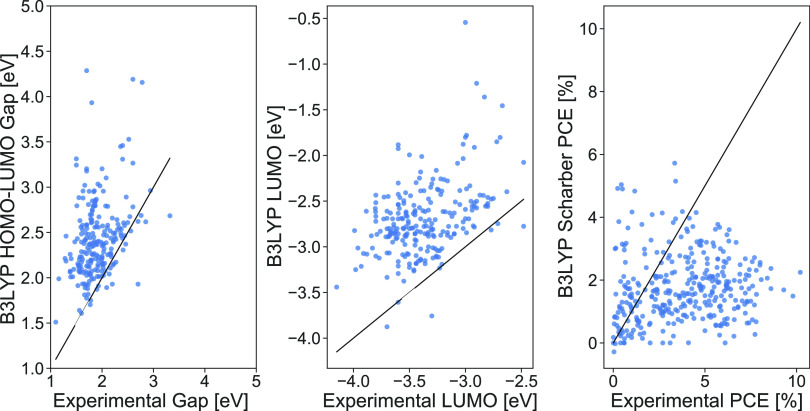
Comparison between the donor gap, donor LUMO energy, and Scharber PCE predicted computationally by DFT and by various experiments in HOPV15.

It is worth noting that the HOPV15 dataset also contains substantial computational data for each of the 350 organic solar cell donor candidates. This includes optimized geometries for up to 20 conformers for each species, which are within 5 kcal/mol of the minimum energy structure at the BP86/def2-SVP level of theory.^22^ Additionally, for each conformer, the HOMO, LUMO, HOMO–LUMO gap, *J*_sc_, *V*_oc_, and Scharber equation PCE are reported having been computed using the def2-SVP basis set and four DFT functionals, namely, BP86, B3LYP, PBE0, and M06-2X. This does provide DFT data that could be incorporated as descriptors in the machine learning model. However, a sample comparison between the HOMO–LUMO gap, LUMO energy, and PCE predicted using B3LYP/def2-SVP and the corresponding experimentally measured values for these properties suggest poor agreement between computation and experiment. This is seen in Figure 1. The agreement is also poor for the other three functionals used. This does suggest that PCEs purely predicted by the Scharber equation are insufficient to predict the actual performance of the material, in agreement with Padula et al.^23^ Furthermore, the plots also suggest that these popular DFT methods struggle when predicting the HOMO–LUMO gap and LUMO energy of the donor, two popular descriptors used for machine learning models of PCE. This may also explain why inclusion of properties computed by DFT in the previous work of Zhao et al.^28^ resulted in only a modest improvement of the ML model performance, in that these properties are not well described by DFT and thus may not correlate well with experimental PCE results. The authors further suggest that while some physical properties predicted through DFT might correlate with PCE, they do not really improve the machine learning performance as they are already correlated strongly with the molecular structure. Given this, it was decided that no additional descriptors predicted by DFT would be included in subsequent machine learning models and that the models would only be trained to relate PCE to the underlying molecular structure of the donor as described by the SMILES strings.

## 3 Methodology

### 3.1 Models and Descriptors Summary

**Figure 2:**
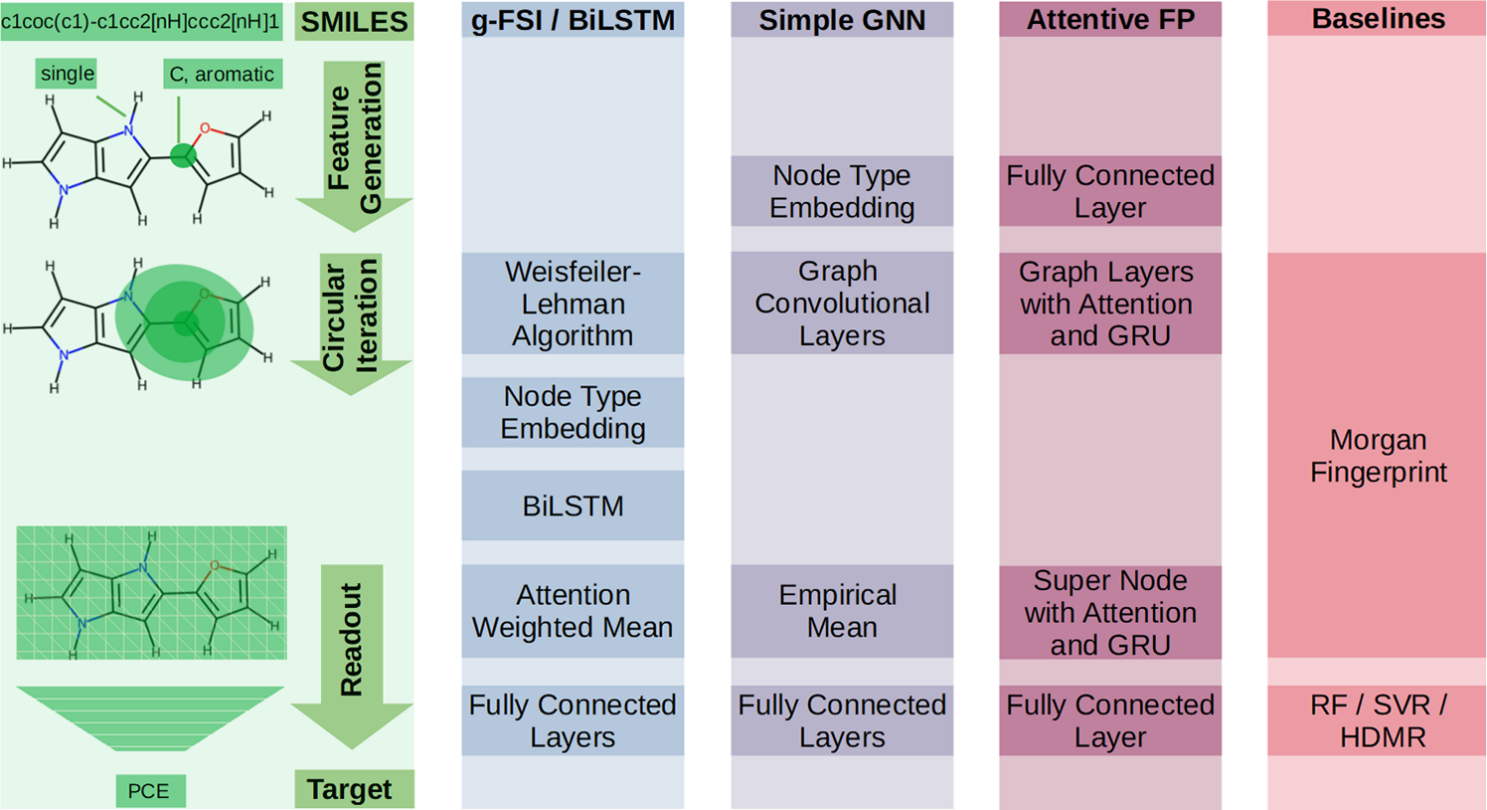
Overview of neural networks and baselines used in this paper and alignment of their building blocks to three general steps: feature generation, circular iteration, and readout.

While the neural networks and ML methods utilized in this paper differ in many respects, they also have some general methodological steps in common. The steps common to all of the ML methods are introduced here, whereas details specific to each ML method are provided in the Supporting Information. An overview of the structure of the ML methods utilized in this work is presented in Figure 2. The left-hand side of Figure 2 shows an example of a SMILES string and its corresponding structure for one of the smaller molecules in the CEPDB. This example structure contains only 21 atoms, including hydrogens, which are treated explicitly throughout this work. Figure 2 also illustrates three general methodological steps in the ML methods: feature generation, circular iteration, and readout. Each of these are discussed in turn below.

#### 3.1.1 Feature Generation

Feature generation is where a SMILES string is converted into a molecular graph. This graph represents the moleculuar structure, with node features including properties such as the atom type and aromaticity and edge features including properties like the bond type. These node and edge features are determined by RDKit.^32^

#### 3.1.2 Circular Iteration

For a given node, circular iteration transforms and joins the features of this node with the features of its neighbor nodes and of the edges that connect them in a circular and iterative manner. An example is illustrated in Figure 2, for the carbon atom marked by a dark green dot. This carbon atom has three direct neighbor nodes with atom types O, C, and C, which are connected by two aromatic bonds, and one single bond. The green circle indicates the merge of the bond and neighbor features for the marked carbon atom in the first iteration. In parallel, the merge is performed analogously for each further node in the molecular graph and its direct neighbors. Afterward, all node states are updated with the merged result. All ML methods perform these transformations and merging, but they do differ in the way of how they transform and merge the information.

Once one iteration is complete, the entire procedure may be repeated once again for the updated node states. After the second iteration, the updated node states now not only include information about the directly neighboring nodes and connections, but also information about the direct neighbors’ neighbors. For the carbon atom in Figure 2, this is indicated by the light green oval covering all nodes with a distance of one or two edges. As a consequence, as more merge and update iterations are performed, each node state will contain information on an increasingly large environment within the molecular graph.

#### 3.1.3 Readout

First, all node states resulting from the previous circular iterations are aggregated to a single state on the graph level (i.e., on the molecule level). Next the aggregated state is used to predict the target value, e.g., by feeding it into a multilayer perceptron. The execution of these two steps comprises readout.

Note that some models in Figure 2 contain additional building blocks or steps that do not align perfectly with these three overarching steps. For instance, the BiLSTM is a central block in the g-FSI/BiLSTM model and is placed between the steps “circular iteration” and “readout”. Detailed descriptions of the steps that are specific to each ML method are provided in the Supporting information.

### 3.2 Training and Hyperparameter Optimization

Given the complexity and the number of different machine learning models considered in this work, performing manual hyperparameter fine-tuning for all of the models would present a substantial challenge. Furthermore, if each model has manual fine-tuning of its hyperparameters, it becomes difficult to create a fair comparison between models, as one may have just had its hyperparameters tuned more carefully than another. Instead, two different automated strategies have been used to try and keep a level of consistency in how the different models have their hyperparameters tuned. These strategies are explained below.

#### 3.2.1 Strategy I

**Figure 3:**
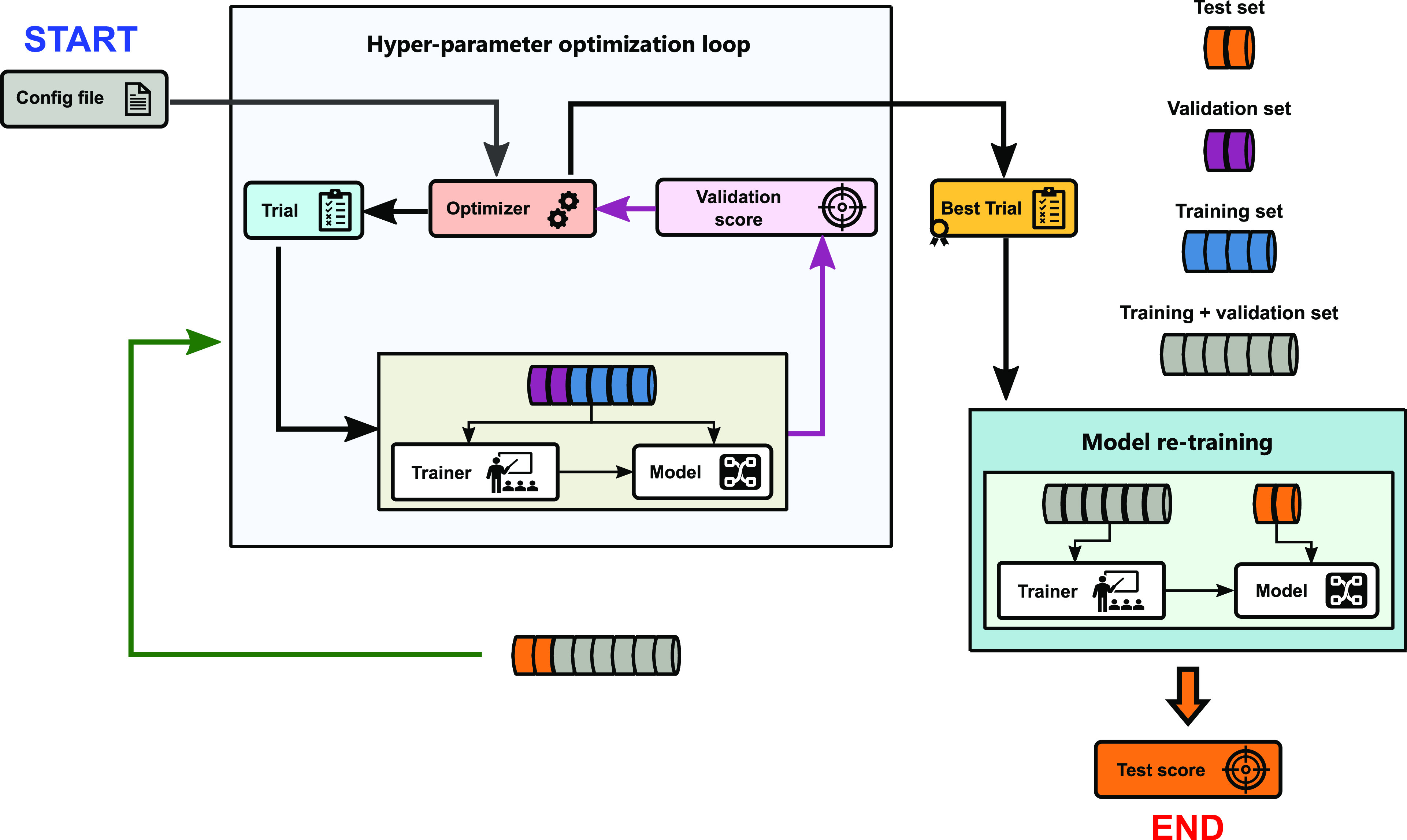
Strategy I: hyperparameter optimization and model re-training.

This strategy is depicted in Figure 3 and has been used for hyperparameter optimization when training each ML method on the CEPDB dataset. The dataset is split into three sets: training, validation, and test sets with a ratio of 0.6/0.2/0.2. Then, a Bayesian optimization-based hyperparameter tuning is performed where the models are trained on the training set with different configurations (trials) sampled within the search space. In the case of the neural models (g-FSI/BiLSTM, simple GNN, and attentive FP), the training process is performed for 400 epochs using Adam Optimizer.^33^ The validation set is used to monitor the training process using the mean squared error as the performance metric. For the neural models, this metric was employed for early stopping to prevent overfitting, as well as assessing the performance of suggested parameter configurations. For all models aside from HDMR, 10 trials are first randomly sampled for the hyperparameter optimizer to estimate a statistical model for the objective function. The remaining 90 trials are suggested by an acquisition function that is continuously updated over the performance of the sampled combination of hyperparameters. The whole process is handled by the tree-structured Parzen estimator algorithm implemented in the Optuna framework.

In the case of the HDMR model, since the only considered hyperparameters are six different fingerprint bit numbers and 4 different radii (as shown in Table S2), it was possible to do a full manual grid-search approach, resulting in 24 trials performed. Subsequently, the hyperparameters of the best trial are used in the final model re-training step. The training and validation sets are combined and reshuffled with a different seed for the new split. Each model was again trained with early stopping monitored. The final model performance is checked on the test set, which is withheld from the machine learning method throughout the hyperparameter optimization process.

#### 3.2.2 Strategy II

**Figure 4:**
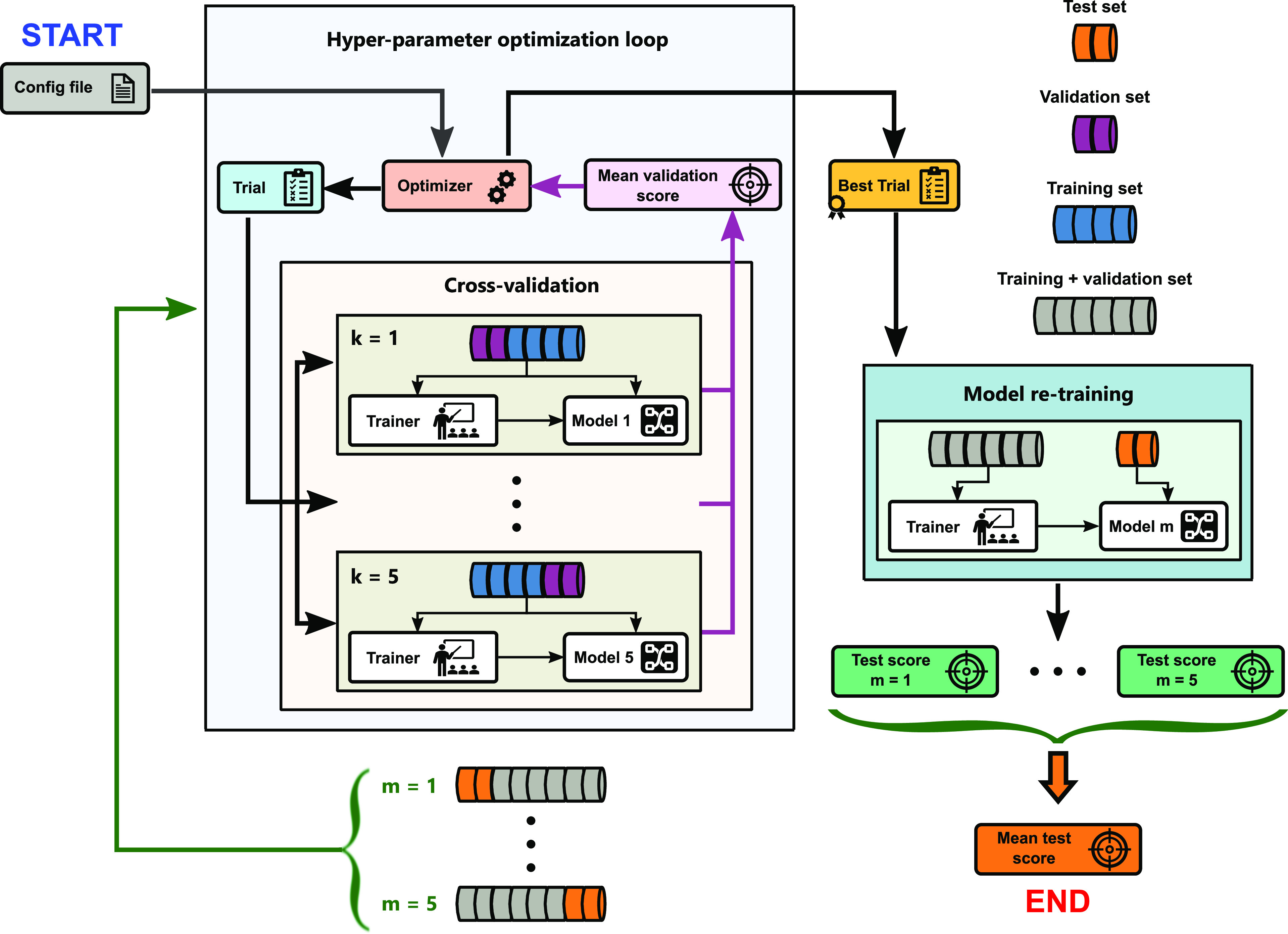
Strategy II: hyperparameter optimization and model re-training.

This strategy is depicted in Figure 4 and has been used to fine-tune the ML models on the HOPV15 dataset. It can be seen that strategy II differs from strategy I only by the additional use of nested cross-validation. In this approach, the hyperparameter optimization and the model generalization error estimation are performed *m* times by separating *m* different test and training sets from the whole dataset. This is also called the outer loop. Then, for each *m*-fold dataset split, the training set is further split into *k* different training and validation sets, which constitutes an inner loop. Subsequently, for the same set of hyperparameters suggested by the optimizer, *k* different models are trained, each using a different *k*-fold split of the total training data. Then, the mean squared validation error across all of the trained models is used to assess the final trial performance. In the end, *m* best trials are found and tested in the model re-training step. The final model test score is taken as the mean squared test error across all *m*-folds. Given that the HOPV15 dataset size is not too prohibitive, the total number of outer and inner folds, (*m*, *k*), was set to 5 for all of the models. The remaining details of the model training and trial sampling procedures are the same as in strategy I.

Further information regarding the choice of hyperparameters set for each model and their sampling ranges can be found in the Supporting Information.

### 3.3 Software and Tools

All of the machine learning models considered in this work, except the HDMR, were implemented in Python. The g-FSI/BiLSTM model was reimplemented using the PyTorch framework and by following the original description of the model by Wu et al.^18^ The PyTorch backend of the Deep Graph Library (DGL)^34^ was used to build the simple GNN model. For attentive FP, the code was obtained from a publicly available repository.^35^ The RF and SVR baseline models were built in Python using the Scikit learn library,^36^ whereas all of the HDMR simulations were performed using a commercial software, Model Development Suite (MoDS).^37^ The conversion of SMILES strings to molecular graphs as well as molecular fingerpint generation were done using the RDKit library.^38^ Finally, the hyperparameter optimizations were performed with Optuna^39^ for all models aside from HDMR.

## 4 Results and Discussion

### 4.1 Harvard Clean Energy Project Dataset Results

This section presents the results obtained by optimizing and training the selected ML models on the CEPDB dataset using strategy I. The final models’ performance metrics for predicting power conversion efficiencies of organic solar cells, in terms of mean squared error (MSE), mean absolute error (MAE), coefficient of determination (*R*^2^), and Pearson correlation coefficient (*r*), are collated in Table 1.

**Table 1:** Performance of the Models Trained on CEPDB Data in Predicting the PCE Values of Organic Photovoltaics

	CEPDB				
model/dataset	set	MSE	MAE	*R*^2^	*r*
g-FSI/BiLSTM	train	0.038	0.151	0.993	0.997
	val	0.207	0.322	0.964	0.982
	test	0.225	0.329	0.961	0.981
simple GNN	train	0.036	0.145	0.994	0.997
	val	0.085	0.208	0.985	0.993
	test	0.091	0.209	0.984	0.992
attentive FP	train	0.024	0.118	0.996	0.998
	val	0.062	0.176	0.989	0.995
	test	0.071	0.180	0.988	0.994
SVR	train	0.008	0.009	0.999	0.999
	test	0.297	0.383	0.949	0.974
RF	train	0.008	0.060	0.999	0.999
	test	0.569	0.534	0.902	0.950
HDMR	train	0.413	0.475	0.929	0.964
	test	0.530	0.536	0.909	0.953

The results in Table 1 suggest that all models can achieve a reasonably good fit for the CEPDB data, with the largest test set MSE being 0.569 for random forests. However, there are still some differences between the performances of the ML methods, and so each method is discussed in turn.

#### 4.1.1 g-FSI/BiLSTM

Although this model still performs rather well with a quite low test set MSE of 0.225, it performs relatively poorly when compared to the other neural models. By comparing the training and validation sets errors, it can be inferred that there is some degree of overfitting. As mentioned previously, this ML architecture has also been applied to the CEPDB dataset by Wu et al.^18^ who managed to obtain the test set MSE of 0.12. Although this is lower than the current results, it is important to understand that the sampled CEPDB data points used in this and Wu et al.^18^ studies were not the same, hence the difference in performance.

#### 4.1.2 Attentive FP

**Figure 5:**
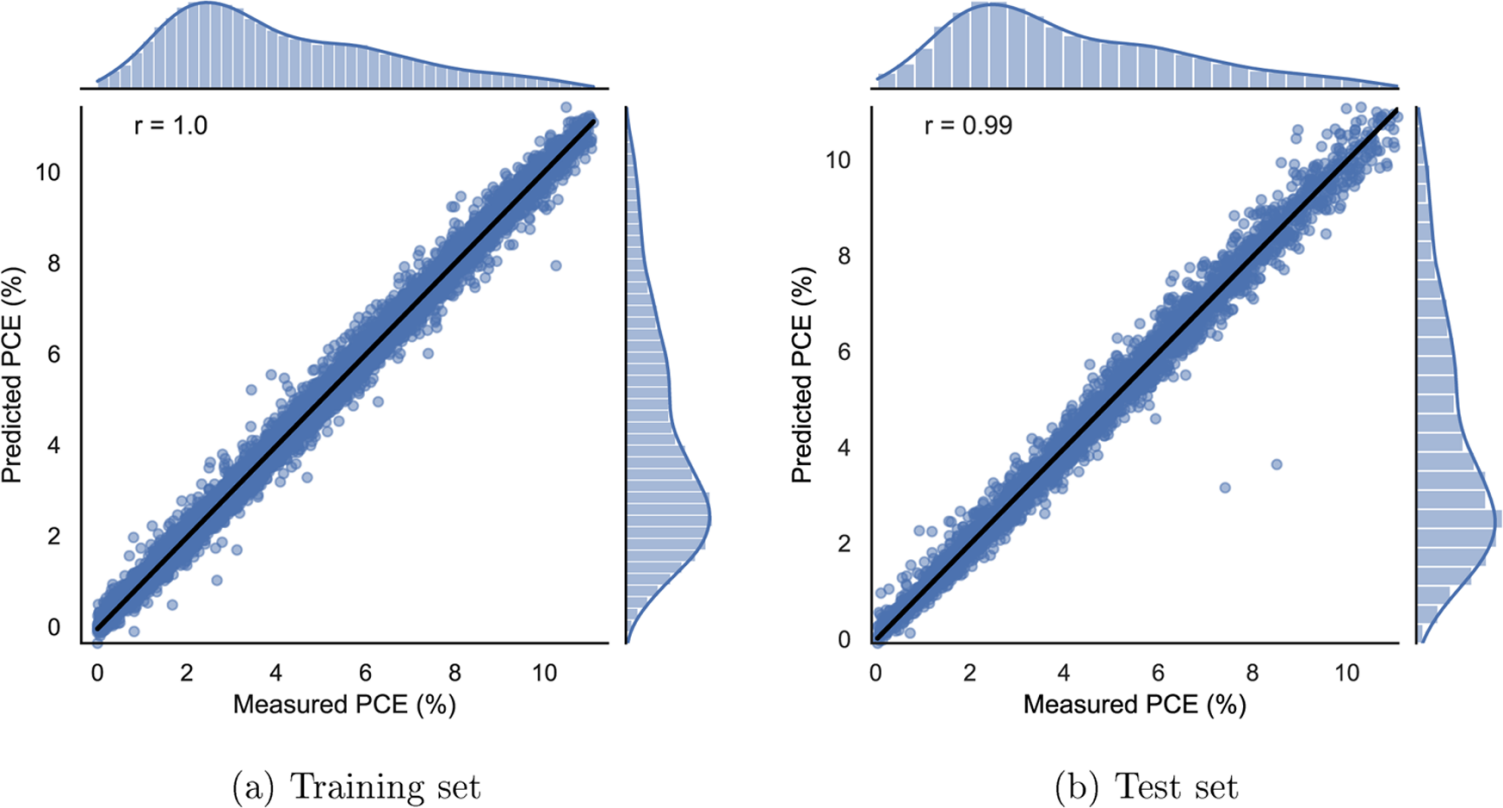
Attentive FP regression plot showing the predicted vs measured PCEs for the training (a) and test (b) sets. The marginal distribution of the measured and predicted PCE values are plotted at the side and on the top.

This model performs the best out of all of the ML methods, with the MSE as low as 0.071 on the test set. This is to the best of our knowledge, the best result obtained so far on the CEPDB dataset. The next best result is from our simple GNN model (explained in the next paragraph), whereas the next best literature result of 0.12 MSE is from Wu et al.,^18^ as explained previously. Figure 5 visualizes the predicted vs measured PCEs for the training and test sets, where only a little scatter is observed. Xiong et al.^15^ evaluated their attentive FP on several datasets ranging from quantum chemistry to physiology and achieved state-of-the-art predictive performance. They also trained their model on a CEPDB subset and reported a mean MSE of 0.82 ± 0.07, which was calculated from three runs for different seeds using optimized hyperparameter values. As described in Section 2, species with a PCE value smaller than 0.0001 were removed from CEPDB during preprocessing. Without the removal of these species, the implementation for attentive FP used in this work yields an error of the same order, suggesting that the results are consistent with those of Xiong et al.^15^

#### 4.1.3 Simple GNN

The model has been found to have the second best performance with the test set MSE of 0.091. This is somewhat surprising, given the simplicity of the method and the fact that it uses only two atom features. This demonstrates the power of graph neural networks, and how they are a rather natural machine learning approach to modeling molecules.

The optimal hyperparameters derived for each neural method are collated in Table S3 in the Supporting Information. It can be seen that the g-FSI/BiLSTM and simple GNN methods have the same optimal embedding dimension and number of MLP hidden layers. The optimal number of neurons in the hidden layer is also nearly the same for these two methods.

#### 4.1.4 Baseline Models

**Figure 6:**
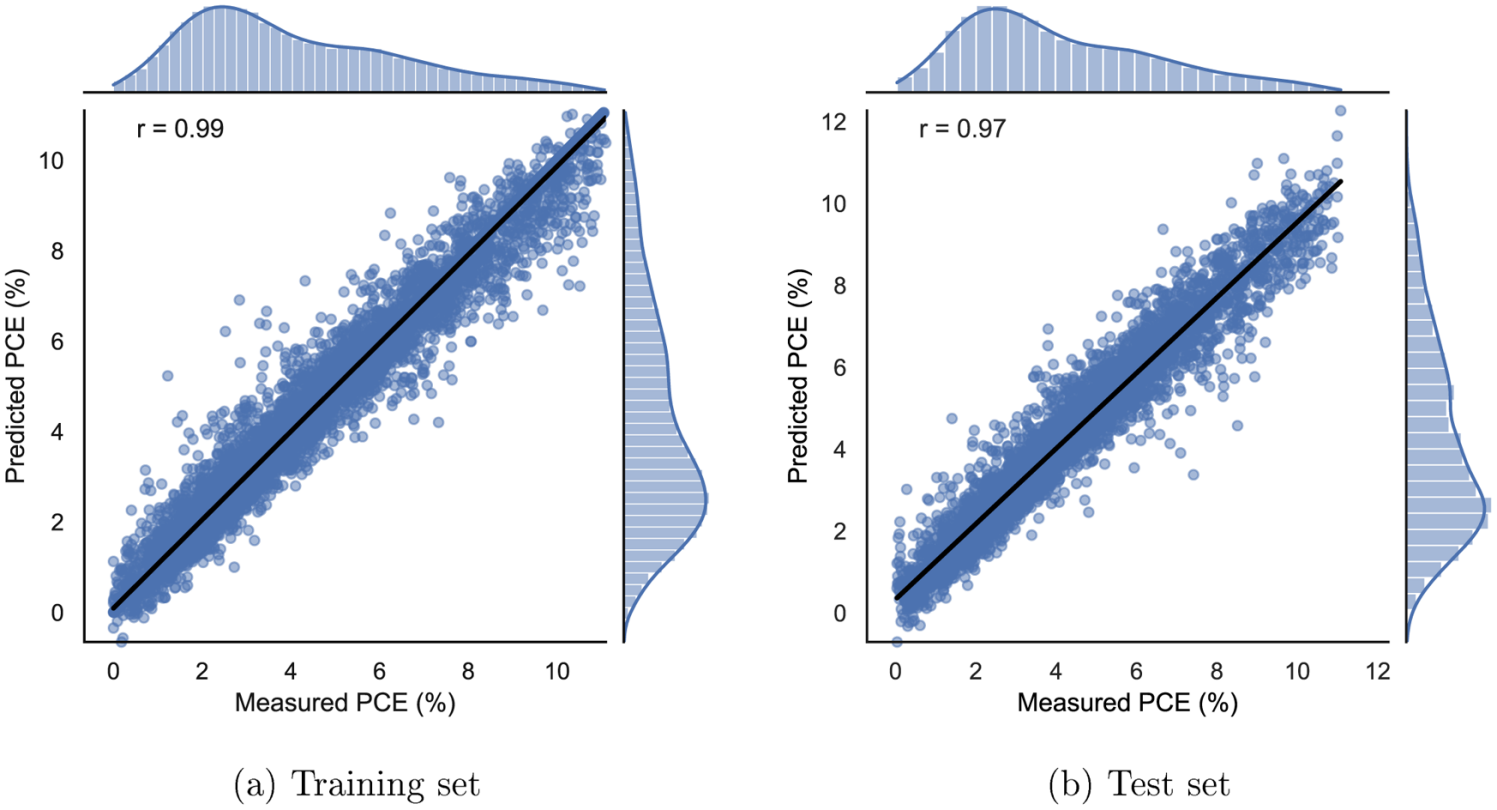
Support vector regression plot showing predicted vs measured PCEs for the training (a) and test (b) sets. The marginal distribution of the measured and predicted PCE values are plotted at the side and on the top.

The three selected baseline models have shown to offer a robust alternative to their neural model counterparts. Support vector regression is the best performing baseline model with a test set MSE of 0.297. Figure 6 shows the predicted vs measured PCEs for the training and test sets. It can be seen that these plots have a bit more scatter in them when compared to the same plots for attentive FP. It can also be noticed from Table 1 that HDMR and random forests have comparable performance with their test set MSEs of 0.530 and 0.569, respectively. This is rather surprising for HDMR, as the model is not naturally suited to deal with integer-valued inputs, making this particular application a rather challenging one.

Table S4 in the Supporting Information lists the most optimal hyperparameters for all of the baseline models. Noticeably, the optimal fingerprint size and radius is same across all three models. This means that there is some generality as to which parameters are suitable for the CEPDB dataset for both the neural and baseline ML methods.

### 4.2 Harvard Organic Photovoltaic Dataset Results

This section presents the results obtained by optimizing and training the selected ML models on the HOPV15 dataset using strategy II. Table 2 collates the mean performance metrics for all of the models across all five outer loop cross-validation folds. The error bar estimates are also provided and are given as one population standard deviation, which for a normal distribution would correspond to a 68% confidence interval.

**Table 2:** Performance of Models Trained on HOPV15 Data in Predicting the PCE Values of Organic Photovoltaics^a^

	HOPV15				
model/dataset	set				*r̅*
g-FSI/BiLSTM	train	1.072 ± 0.675	0.780 ± 0.271	0.776 ± 0.149	0.900 ± 0.079
	val	3.273 ± 0.447	1.425 ± 0.141	0.363 ± 0.067	0.625 ± 0.052
	test	3.486 ± 0.647	1.480 ± 0.169	0.299 ± 0.090	0.580 ± 0.064
simple GNN	train	1.494 ± 0.712	0.918 ± 0.300	0.711 ± 0.128	0.866 ± 0.065
	val	2.641 ± 0.695	1.293 ± 0.166	0.426 ± 0.073	0.680 ± 0.063
	test	3.295 ± 0.279	1.454 ± 0.092	0.330 ± 0.053	0.598 ± 0.047
attentive FP	train	2.936 ± 1.101	1.377 ± 0.377	0.420 ± 0.211	0.648 ± 0.138
	val	3.020 ± 0.964	1.397 ± 0.227	0.355 ± 0.081	0.597 ± 0.070
	test	4.417 ± 1.503	1.672 ± 0.223	0.127 ± 0.193	0.455 ± 0.113
SVR	train	0.276 ± 0.479	0.196 ± 0.306	0.946 ± 0.093	0.973 ± 0.046
	test	2.687 ± 0.487	1.319 ± 0.095	0.453 ± 0.109	0.684 ± 0.083
RF	train	0.703 ± 0.610	0.579 ± 0.343	0.859 ± 0.123	0.934 ± 0.059
	test	2.876 ± 0.415	1.318 ± 0.065	0.414 ± 0.089	0.657 ± 0.061
HDMR	train	0.724 ± 0.171	0.673 ± 0.070	0.855 ± 0.035	0.927 ± 0.019
	test	3.185 ± 0.540	1.411 ± 0.080	0.350 ± 0.135	0.623 ± 0.078

a Provided model accuracy metrics are given as a mean across all *m*-folds and the error bars are given as σ, which for a normal distribution would correspond to a confidence level of 68%.

#### 4.2.1 g-FSI/BiLSTM

The model mean test MSE is the second lowest of the neural models. Nevertheless, the model performance (MSE) is much worse compared with the CEPDB dataset. The obtained mean test MAE of 1.480 is a bit higher than the value reported by Wu et al.,^18^ where it was about 1.25 (without cross-validation).

#### 4.2.2 Attentive FP

This model now has the highest MSE of 4.417 across all of the tried models. The model also has the largest variation across the outer cross-validation folds, meaning it is very sensitive to the partition of the training set. This is in complete contrast to its performance on the CEPDB dataset, when it was found to be the best performer. A potential explanation for these “surprising” findings could be that among all of the ML models, attentive FP is the most sophisticated one. Therefore, the small and varied amount of data in the HOPV15 dataset might make it difficult to train.

#### 4.2.3 Simple GNN

Although the model has the best test MSE and MAE among all of the tried neural models and one of the lowest variations across outer cross-validation folds, its performance on HOPV15 is still much worse compared to CEPDB.

#### 4.2.4 Baseline Models

In the case of the HOPV15 dataset, the three baseline models have smaller MSE than their neural counterparts. This time, SVR is not only the best performing baseline model with respect to the test MSE, as it was on the CEPDB dataset, but it is also the best performing overall model. The second best performing model is RF with the test set MSE of 2.876 followed by the HDMR model with 3.185 MSE. However, the mean test errors for SVR, RF, and HDMR only slightly differ, in particular when considering their relatively large empirical variance. Nevertheless, the baseline model results confirm the known fact that when training to smaller datasets with larger variability, it can be advantageous to have fewer degrees of freedom in the models.

As the models generally performed poorly on the HOPV15 dataset, the potential of transfer learning to improve model performance was also assessed. Transfer learning was implemented for all three neural network models by taking the best model identified during the hyperparameter optimization on the CEPDB dataset and continually training on the HOPV15 dataset in an end-to-end fashion. The rationale is that the neural network models are believed to be able to generalize the fragments and substructures of a molecule that are important to PCE when trained on the CEPDB, thus accelerating the learning process when the model is applied to a smaller but somewhat different dataset like HOPV15. The model performance when utilizing transfer learning was compared to the model performance when the weights were randomly initialized and trained on the HOPV15 dataset. However, the transfer learning did not result in any statistically significant improvement in performance.

Analyzing the results it can be seen that for the HOPV15 dataset, the selected machine learning models are all unable to present a clear correlation between the molecular structures from SMILES and the target PCE, which was not the case for the CEPDB dataset. It is then important to discuss potential causes of such a poor model behaviour on HOPV15.

One clear difference between the CEPDB and HOPV15 datasets is that the HOPV15 dataset is substantially smaller. To explore this, learning curves were constructed using strategy II and the random forest baseline model, by training to 40, 60, 80, and 100% of the HOPV15 dataset. However, as detailed in Supporting Information Figure S1, no clear correlation could be found between the sample size and the MSE. This would suggest that additional reasons other than the sample size should also be considered.

Another, rather obvious, reason is that the HOPV15 data are inhomogeneous, meaning that they were collated across different labs, so it is very unlikely that the data points are from the same experimental setups. As a consequence, the PCEs are much more difficult to compare, as they are not all determined using the same method as is done computationally with the CEPDB. Different experiments also have different associated errors with them, which have not been included or taken into consideration here. Furthermore, there is an extra atom type included in the HOPV15 that is not in the CEPDB, which is fluorine. The number of fragment types in HOPV15 is also much larger in comparison to CEPDB, with HOPV15 having 156 fragments in comparison to just 56 in CEPDB. This means that the chemical complexity of HOPV15 is higher than CEPDB, which poses a challenge as the dataset is much smaller to begin with. Additionally, the number of variables influencing PCE in the real-world OPV materials is likely larger compared to the number of variables in the simple Scharber model (CEPDB dataset). While some of these variables may correlate with structural information encoded in SMILES strings, it is plausible that there are other nonaccounted for factors. For example, this includes bulk properties of OPV materials such as the structure of the layer and OPV itself, the microstructure of any polymers used in the OPV conjunction, and the contact area between the donor and acceptor in the OPV to name a few.^40^ These factors likely make the HOPV15 dataset more challenging for machine learning purposes.

## 5 Conclusions

In this paper, the ability of five machine learning models and HDMR to predict the PCE of organic photovoltaics based on molecular structure information is assessed, including the impact and implications of the choice of training data. Three neural (gFSI/BiLSTM, simple GNN, and attentive FP) and three baseline (SVR, RF, and HDMR) models are trained on the larger, computational Harvard CEPDB dataset and on the much smaller, experimental HOPV15 dataset.

The contrasting datasets result in contrasting performance of the machine learning models. In the case of the CEPDB, the simple GNN and attentive FP neural models work very well, and the attentive FP in particular achieves very low test MSE. The g-FSI/BiLSTM performs noticeably worse. The baseline models perform worse on average than the neural models, although SVR does reasonably well. In general, all of the machine learning models are able to derive high correlation coefficients between the learned PCE values and the actual PCE values in the CEPDB, suggesting that the CEPDB PCE values correlate well with the SMILES string of the donor molecules.

In the case of the HOPV15, the performance of all machine learning models is much worse. Attentive FP, in terms of test set MSE, now performs the worst, with simple GNN and g-FSI/BiLSTM also presenting very large MSE errors. Contrary to the CEPDB, the baseline models now outperform the neural methods, which could be due to the fact that the neural methods need to train the weights and have insufficient data to do so. Still, the performance of all machine learning models is not very good. This is likely due to the nature of the HOPV15 dataset, which is smaller and also much less homogeneous than that of CEPDB due to expected differences in experimental setups, larger chemical complexity of the species in the dataset, and possibly a larger number of variables influencing real-world organic solar cell PCEs that may not be strongly correlated with the structural information of the donor molecules encoded in the SMILES strings, such as bulk solar cell properties. Transfer learning was also tried for the neural models by first training on CEPDB and then training on the HOPV15 dataset. The transfer learning did not result in any statistically significant changes in performance, which is possibly due to the aforementioned differences between the two datasets.

Ultimately, while a variety of machine learning methods can accurately model PCEs predicted by the Scharber model and DFT, they struggle with modelling experimentally determined PCEs. This is an issue as the computed PCEs do not match well with experimentally determined PCEs. Going forward, to improve the performance of ML models in predicting PCEs that agree with experimental methods, more experimental measurements at a consistent set of experimental conditions would be useful. Alternatively, trying to improve the computational results so that they are more in line with experimental measurements, either by making use of more accurate quantum chemical calculations, or better methods for estimating the PCE, may also help, as these are much easier to standardize and a large amount of starting data that can be improved upon already exists. A suggestion could be to investigate which computational chemical methods can give predictions of the physical properties of interest (like the HOMO–LUMO gap) that are in line with experimental measurements. This will hopefully improve the potential of fast, computational screening of candidate organic photovoltaic donors for clean energy generation in the future.

## Supporting Information Available

Models’ description; models’ hyperparameters; and random forests HOPV15 learning curve (PDF)

## References

[ref1] Snaith H. J. Perovskites: the emergence of a new era for low-cost, high-efficiency solar cells. J. Phys. Chem. Lett. 2013, 4, 3623–3630. 10.1021/jz4020162.

[ref2] Green M. A.; Ho-Baillie A.; Snaith H. J. The emergence of perovskite solar cells. Nat. Photonics 2014, 8, 506–514. 10.1038/nphoton.2014.134.

[ref3] Jeong M.; Choi I. W.; Go E. M.; Cho Y.; Kim M.; Lee B.; Jeong S.; Jo Y.; Choi H. W.; Lee J.; et al. Stable perovskite solar cells with efficiency exceeding 24.8% and 0.3-V voltage loss. Science 2020, 369, 1615–1620. 10.1126/science.abb7167. 32973026

[ref4] Urbina A. The balance between efficiency, stability and environmental impacts in perovskite solar cells: a review. J. Phys.: Energy 2020, 2, 022001 10.1088/2515-7655/ab5eee.

[ref5] Wöhrle D.; Meissner D. Organic solar cells. Adv. Mater. 1991, 3, 129–138. 10.1002/adma.19910030303.

[ref6] Scharber M. C.; Mühlbacher D.; Koppe M.; Denk P.; Waldauf C.; Heeger A. J.; Brabec C. J. Design rules for donors in bulk-heterojunction solar cells–Towards 10% energy-conversion efficiency. Adv. Mater. 2006, 18, 789–794. 10.1002/adma.200501717.

[ref7] Abdulrazzaq O. A.; Saini V.; Bourdo S.; Dervishi E.; Biris A. S. Organic solar cells: a review of materials, limitations, and possibilities for improvement. Part. Sci. Technol. 2013, 31, 427–442. 10.1080/02726351.2013.769470.

[ref8] Hachmann J.; Olivares-Amaya R.; Atahan-Evrenk S.; Amador-Bedolla C.; Sánchez-Carrera R. S.; Gold-Parker A.; Vogt L.; Brockway A. M.; Aspuru-Guzik A. The Harvard clean energy project: large-scale computational screening and design of organic photovoltaics on the world community grid. J. Phys. Chem. Lett. 2011, 2, 2241–2251. 10.1021/jz200866s.

[ref9] Scharber M. C.; Sariciftci N. S. Efficiency of bulk-heterojunction organic solar cells. Prog. Polym. Sci. 2013, 38, 1929–1940. 10.1016/j.progpolymsci.2013.05.001. 24302787 PMC3837184

[ref10] Rühle S. Tabulated values of the Shockley-Queisser limit for single junction solar cells. Sol. Energy 2016, 130, 139–147. 10.1016/j.solener.2016.02.015.

[ref11] Xie Y.; Wang W.; Huang W.; Lin F.; Li T.; Liu S.; Zhan X.; Liang Y.; Gao C.; Wu H.; et al. Assessing the energy offset at the electron donor/acceptor interface in organic solar cells through radiative efficiency measurements. Energy Environ. Sci. 2019, 12, 3556–3566. 10.1039/C9EE02939G.

[ref12] Pyzer-Knapp E. O.; Li K.; Aspuru-Guzik A. Learning from the Harvard clean energy project: The use of neural networks to accelerate materials discovery. Adv. Funct. Mater. 2015, 25, 6495–6502. 10.1002/adfm.201501919.

[ref13] Sun W.; Li M.; Li Y.; Wu Z.; Sun Y.; Lu S.; Xiao Z.; Zhao B.; Sun K. The use of deep learning to fast evaluate organic photovoltaic materials. Adv. Theory Simul. 2019, 2, 1800116 10.1002/adts.201800116.

[ref14] Duvenaud D. K.; Maclaurin D.; Iparraguirre J.; Bombarell R.; Hirzel T.; Aspuru-Guzik A.; Adams R. P. Convolutional networks on graphs for learning molecular fingerprints. Adv. Neural Inf. Process. Syst. 2015, 28, 2224–2232.

[ref15] Xiong Z.; Wang D.; Liu X.; Zhong F.; Wan X.; Li X.; Li Z.; Luo X.; Chen K.; Jiang H.; et al. Pushing the boundaries of molecular representation for drug discovery with the graph attention mechanism. J. Med. Chem. 2020, 8749–8760. 10.1021/acs.jmedchem.9b00959. 31408336

[ref16] Ryu S.; Lim J.; Hong S. H.; Kim W. Y. Deeply learning molecular structure-property relationships using attention-and gate-augmented graph convolutional network. 2018, arXiv:1805.10988. arXiv.org e-Print archive. https://arxiv.org/abs/1805.10988 (accessed May 28, 2018).

[ref17] Bahdanau D.; Cho K.; Bengio Y. Neural machine translation by jointly learning to align and translate. 2014, arXiv:1409.0473. arXiv.org e-Print archive. https://arxiv.org/abs/1409.0473 (accessed Sep 1, 2014).

[ref18] Wu J.; Wang S.; Zhou L.; Ji X.; Dai Y.; Dang Y.; Kraft M. Deep-Learning Architecture in QSPR Modeling for the Prediction of Energy Conversion Efficiency of Solar Cells. Ind. Eng. Chem. Res. 2020, 18991–19000. 10.1021/acs.iecr.0c03880.

[ref19] Hochreiter S.; Schmidhuber J. Long short-term memory. Neural Comput. 1997, 9, 1735–1780. 10.1162/neco.1997.9.8.1735. 9377276

[ref20] Lopez S. A.; Sanchez-Lengeling B.; de Goes Soares J.; Aspuru-Guzik A. Design principles and top non-fullerene acceptor candidates for organic photovoltaics. Joule 2017, 1, 857–870. 10.1016/j.joule.2017.10.006.

[ref21] Pyzer-Knapp E. O.; Simm G. N.; Guzik A. A. A Bayesian approach to calibrating high-throughput virtual screening results and application to organic photovoltaic materials. Mater. Horiz. 2016, 3, 226–233. 10.1039/C5MH00282F.

[ref22] Lopez S. A.; Pyzer-Knapp E. O.; Simm G. N.; Lutzow T.; Li K.; Seress L. R.; Hachmann J.; Aspuru-Guzik A. The Harvard organic photovoltaic dataset. Sci. Data 2016, 3, 160086 10.1038/sdata.2016.86. 27676312 PMC5037972

[ref23] Padula D.; Simpson J. D.; Troisi A. Combining electronic and structural features in machine learning models to predict organic solar cells properties. Mater. Horiz. 2019, 6, 343–349. 10.1039/C8MH01135D.

[ref24] Nagasawa S.; Al-Naamani E.; Saeki A. Computer-aided screening of conjugated polymers for organic solar cell: classification by random forest. J. Phys. Chem. Lett. 2018, 9, 2639–2646. 10.1021/acs.jpclett.8b00635. 29733216

[ref25] Sun W.; Zheng Y.; Yang K.; Zhang Q.; Shah A. A.; Wu Z.; Sun Y.; Feng L.; Chen D.; Xiao Z.; et al. Machine learning–assisted molecular design and efficiency prediction for high-performance organic photovoltaic materials. Sci. Adv. 2019, 5, eaay4275 10.1126/sciadv.aay4275. 31723607 PMC6839938

[ref26] Sahu H.; Yang F.; Ye X.; Ma J.; Fang W.; Ma H. Designing promising molecules for organic solar cells via machine learning assisted virtual screening. J. Mater. Chem. A 2019, 7, 17480–17488. 10.1039/C9TA04097H.

[ref27] Sahu H.; Ma H. Unraveling correlations between molecular properties and device parameters of organic solar cells using machine learning. J. Phys. Chem. Lett. 2019, 10, 7277–7284. 10.1021/acs.jpclett.9b02772. 31702163

[ref28] Zhao Z.-W.; del Cueto M.; Geng Y.; Troisi A. Effect of Increasing the Descriptor Set on Machine Learning Prediction of Small Molecule-Based Organic Solar Cells. Chem. Mater. 2020, 32, 7777–7787. 10.1021/acs.chemmater.0c02325.

[ref29] Meftahi N.; Klymenko M.; Christofferson A. J.; Bach U.; Winkler D. A.; Russo S. P. Machine learning property prediction for organic photovoltaic devices. npj Comput. Mater. 2020, 6, 166 10.1038/s41524-020-00429-w.

[ref30] Xu Z.; Chen L.-M.; Chen M.-H.; Li G.; Yang Y. Energy level alignment of poly (3-hexylthiophene):[6, 6]-phenyl C 61 butyric acid methyl ester bulk heterojunction. Appl. Phys. Lett. 2009, 95, 178.

[ref31] Gueymard C. A.; Myers D.; Emery K. Proposed reference irradiance spectra for solar energy systems testing. Sol. Energy 2002, 73, 443–467. 10.1016/S0038-092X(03)00005-7.

[ref32] Landrum G. RDKit: A Software Suite for Cheminformatics, Computational Chemistry, and Predictive Modeling, 2013.

[ref33] Kingma D. P.; Ba J. Adam: A Method for Stochastic Optimization. 2017, arXiv:1412.6980. arXiv.org e-Print archive. https://arxiv.org/abs/1412.6980 (accessed Dec 22, 2014).

[ref34] Wang M.; Zheng D.; Ye Z.; Gan Q.; Li M.; Song X.; Zhou J.; Ma C.; Yu L.; Gai Y.; Xiao T.; He T.; Karypis G.; Li J.; Zhang Z. Deep Graph Library: A Graph-Centric, Highly-Performant Package for Graph Neural Networks. 2019, arXiv:1909.01315. arXiv.org e-Print archive. https://arxiv.org/abs/1909.01315 (accessed Sep 3, 2019).

[ref35] DGL-LifeSci. https://github.com/awslabs/dgl-lifesci (accessed Dec 16, 2020).

[ref36] Pedregosa F.; et al. Scikit-learn: Machine Learning in Python. J. Mach. Learn. Res. 2011, 12, 2825–2830.

[ref37] CMCL Innovations, MoDS (Model Development Suite), Version 2020.2.2. 2020. https://cmclinnovations.com/products/mods/.

[ref38] RDKit: Open-source cheminformatics. https://www.rdkit.org (accessed Dec 16, 2020).

[ref39] Akiba T.; Sano S.; Yanase T.; Ohta T.; Koyama M. In Optuna: A Next-generation Hyperparameter Optimization Framework, Proceedings of the 25rd ACM SIGKDD International Conference on Knowledge Discovery and Data Mining, 2019.

[ref40] Vandewal K.; Himmelberger S.; Salleo A. Structural factors that affect the performance of organic bulk heterojunction solar cells. Macromolecules 2013, 46, 6379–6387. 10.1021/ma400924b.

