# Supplementary material for: Predicting Power Conversion Efficiency of Organic Photovoltaics: Models and Data Analysis
Source: ACS Omega. 2021 Sep 6;6(37):23764–75. doi: 10.1021/acsomega.1c02156 (PMC8459373; doi:10.1021/acsomega.1c02156)
Supplement: Supplementary file 1 — ao1c02156_si_001.pdf [file ao1c02156_si_001.pdf]

Predicting power conversion efficiency of organic photovoltaics: models and data analysis

Andreas Eibeck,[†] Daniel Nurkowski,[‡] Angiras Menon,[¶] Jiaru Bai,[¶] Jinkui Wu,[§] Li Zhou,[§] Sebastian Mosbach,[‡,¶] Jethro Akroyd,[‡,¶] and Markus Kraft[*,¶,†,‖]

†*CARES, Cambridge Centre for Advanced Research and Education in Singapore, 1 Create Way, CREATE Tower, #05-05, Singapore, 138602*

‡*CMCL Innovations, Sheraton House, Castle Park, Cambridge CB3 0AX, UK*

¶*Department of Chemical Engineering and Biotechnology, University of Cambridge, Philippa Fawcett Drive, Cambridge, CB3 0AS, UK*

§*School of Chemical Engineering, Sichuan University Sichuan, China*

‖*School of Chemical and Biomedical Engineering, Nanyang Technological University, 62 Nanyang Drive, Singapore, 637459*

E-mail: mk306@cam.ac.uk

S1 Models description

S1.1 Bi-directional long short-term memory network

A sequential and deep-learning model using a bi-directional long short-term memory network with an attention mechanism and multilayer perceptron has been implemented. This architecture has been proven to be very successful for organic photovoltaics PCE prediction[1],

thus it was decided to use it in this work as one of the neural models. Only the key model aspects are provided here. Readers are directed to[1,2] for a more detailed description.

Feature generation. This step follows the common procedure as described previously. Atom types and their aromaticity are used to encode the node features whereas bond types (single, double, triple and aromatic bonds) are used to encode the edge features.

Circular iteration. The Weisfeiler-Lehman algorithm[3] is used to circulate around the nodes of molecular graphs generated in the previous step. The algorithm consists of two stages: (i) an initial assignment stage, in which each node and edge are assigned the unique integer identifiers that represent their starting features set, and (ii) an iterative updating stage, in which each node identifier is updated to reflect the identifiers of its neighbours and each edge identifier is updated to reflect the identifiers of nodes it connects. Once all nodes and edges have generated their new identifiers, the algorithm cycle is repeated until the requested number of iterations is reached. At the end, the final node identifiers are assembled into the final molecular fingerprint vector. Since the node and edge features only include atom and bond types information, one can also think about this molecular fingerprint vector as a unique list of sub-molecular fragments building the parent molecule, hence the name graph fragments sequence identifiers (g-FSI) in the original publication[1]. In this paper only a single iteration of the Weisfeiler-Lehman algorithm has been performed by setting its radius parameter to one. To avoid the confusion with ordinary BiLSTM networks, the model will be referred to as g-FSI/BiLSTM for the remainder of the paper.

The g-FSI vector encodes two important structural aspects: fragment (fingerprint) types and their order in the molecule. Note that mapping discovered fragment types to unique integer identifiers proceeds on a first-found first-served basis with no hashing. Therefore, it is dependent on the order in which the data is processed. In order to eliminate this dependency, prior to using the model, the selected datasets are processed first to create the fragment-identifiers look-up dictionaries. This step also finds the molecules with the largest

number of fragments which are in turn used to define the maximum sequence length for the BiLSTM network. Furthermore, it is important to highlight that the order of fragments in the g-FSI vector depends on the order in which the molecular graph nodes are processed. To keep the fragments that are spatially close to each other together (e.g two fragments that are part of the same aromatic ring), the molecular graph has been iterated in the breadth first search (BFS) order.

Node type embedding and BiLSTM. This step starts from passing the g-FSI vectors through a trainable embedding layer, which for a single molecule, results in creating a sequence of real-valued fragments embedding vectors (one for each fragment integer identifier). Secondly, the embedding vectors are processed by the forward and backward BiLSTM cells resulting in a sequence of hidden forward and backward state vectors.

Readout. In order to obtain the final PCE value, the two BiLSTM hidden states are concatenated and weighted-summed in the attention layer. This is followed by propagating the attention layer output through a multilayer perceptron.

S1.2 Simple graph neural network

The neural network presented in this section is simple in three respects: (1) It belongs to the class of graph convolutional networks but is implemented in a straight-forward manner. Compared to[4] and[5] it simplifies the iteration update by applying the empirical mean. (2) Moreover, compared to g-FSI/BiLSTM model and Attentive FP, it does not make use of any attention mechanism. That means it treats all neighbours of all nodes in the same way. (3) It uses only a small number of node types as features and no edge features.

Feature generation. All distinguishable tuples of the form (atom type, atom aromaticity) are identified in the datasets leading to 8 and 12 different node types for CEPDB and HOPV15. This approach is equivalent to the Weisfeiler-Lehman algorithm for radius zero.

Then, each different node type is mapped to its own vector in the space $\mathbb{R}^{\mathrm{ED}}$ where ED denotes the embedding dimension and the vector elements are trainable weights. While the node types for Simple GNN are different from the fragment types for the g-FSI/BiLSTM model, their embedding into a continuous space is analogous.

Circular iteration. For each node v in the molecular graph, the embedding vector is chosen corresponding to its node type and used as initial node state $h^0(v) \in \mathbb{R}^{\mathrm{ED}}$. The graph layers of simple GNN are indexed with l ranging from 1 to the overall layer count, CL. Each layer can be described in terms of the message passing neural network (MPNN) framework introduced in:[6] First, messages with transformed states are received from v's direct neighbour nodes $N(v)$ and aggregated,

$$m^l(v) = \frac{1}{\#N(v)} \sum_{u \in N(v)} W^l h^{l-1}(u) \tag{S1}$$

where $W^l \in \mathbb{R}^{\mathrm{ED} \times \mathrm{ED}}$ denotes a matrix of trainable weights and $\#N(v)$ is the number of neighbour nodes. Finally, the state of node v at layer l is updated by

$$h^l(v) = \mathrm{relu}(h^{l-1}(v) + m^l(v)) \tag{S2}$$

where the rectified linear activation function, relu, is applied component-wise. For simplicity, W^l is a square matrix such that all node states have the same dimension, ED, throughout all graph layers. Here, the layer count, CL, plays the same role as for Attentive FP, as the radius in the Weisfeiler-Lehman algorithm, and as the radius for generating Morgan fingerprints: it corresponds to the maximum distance of (direct and indirect) neighbour nodes that may contribute to the final state of node v.

Readout. The state of the graph G is set to the empirical mean over all node states at layer $l = \mathrm{CL}$, i.e.

$$h(G) = \frac{1}{\#N(G)} \sum_{u \in N(G)} h^{\mathrm{CL}}(u) \tag{S3}$$

where $N(G)$ is the set of all nodes in G. Finally, the graph state $h(G) \in \mathbb{R}^{\mathrm{ED}}$ is fed into a multilayer perceptron with one output neuron for PCE prediction analogously to the g-FSI/BiLSTM model.

S1.3 Attentive fingerprint

Attentive FP (fingerprint) is a graph neural network that was introduced by Xiong et al.[7] and achieves state-of-the-art performance on several data sets that are widely used in ML research according to a comprehensive assessment of several neural networks and ML methods by Wu et al.[8]. Attentive FP makes use both of Gated Recurrent Units (GRU,[9]) and attention and thus resumes ideas of Gated Graph Neural Networks[10] and Graph Attention Networks.[11] It applies the attention mechanism both on the atomic node level and on the molecular graph level to focus on substructures that are relevant for predicting target variables.

Feature generation. Nine node features (including atom types and atom aromaticity) and four edge features (including bond type) are used as input features, with most of them being one-hot encoded. A detailed list of the input features can be found in Xiong et al.[7].

Circular iteration. For each target node v in the molecular graph, its node features are fed into a fully connected layer with FS units where FS denotes the Fingerprint Size. The output is used as initial node state $h^0(v) \in \mathbb{R}^{\mathrm{FS}}$. Edge features are handled explicitly only at the beginning of the first iteration: the node features of each direct neighbour $u \in N(v)$ and the features of the edge between u and v are concatenated and fed into another fully connected layer having the same number FS of units. From here, all states have the same size FS and the actual circular iteration can be formulated in terms of the MPNN framework[6] - analogously to the simple GNN from the previous section. Roughly speaking, all summands

in Equation S1 are multiplied with their corresponding attention weights calculated between the states of v and u and replacing the uniform weights $\frac{1}{\#N(v)}$. Then, their sum is fed as input vector into a GRU which replaces the simple update function in Equation S2. This means the state of v is updated to the resulting hidden state of the GRU at the end of each iteration and is used as the starting point for the next iteration.

Readout. A virtual super node is added to the molecular graph and connected to all of its atom nodes. Its state represents the state of the entire graph and is initialized with the sum of all atom node states updated at the end of the last iteration. The super node state itself is updated throughout several iterations using its own GRU. In each iteration, the attention weight between the super node and an atom node is calculated in the same way as between two atom nodes. Finally, the super node state is fed into a fully connected layer which has one output neuron in the case of PCE as target variable.

The GRU can be regarded as a less complex version of an LSTM. The g-FSI/BiLSTM model uses breadth first search for ordering the fragments of a molecule and applies two LSTMs in a bidirectional manner to find patterns within the "horizontal" sequence of ordered fragments. In contrast, the GRU in Attentive FP conveys information for each target node in the molecular graph "vertically", i.e. from layer to layer, and in each layer, the local environment from its direct neighbours is merged with its own information from previous layers (i.e. from previous time steps). Moreover, the information flows for all nodes layer-wise and in parallel and the nodes share the same GRU and corresponding trainable weights.

S1.4 Baseline models

In this paper, we use Morgan fingerprints as input features for all baseline methods. The main idea behind Morgan fingerprints stems from the Morgan algorithm[12] which aims to provide a unique numbering scheme of chemical structures. The Morgan algorithm is specific to molecular graphs and tries to solve the isomorphism problem between them, while the

Weisfeiler-Lehman algorithm shares the same goal for graphs in general.

RDKit implements Morgan fingerprints along the Extended Connectivity Fingerprint algorithm, a modified Morgan algorithm described in.[13] The generation of Morgan fingerprints requires two integer-valued parameters - the fingerprint radius, FR, and the fingerprint size, FS, - and involves several steps as illustrated in Figure 2. This includes applying the three general steps of feature generation, circular iteration, and readout as follows:

Feature Generation. RDKit converts SMILES into molecular graphs with atom and bond features and uses these features to generate the corresponding Morgan fingerprints.

Circular iteration. For each atom in the molecular graph, an identifier is initialized as a bit vector derived from the atom features. In a predefined order, each identifier is concatenated with the identifiers attached to its direct neighbour atoms and with the bit-encoded types of bonds in between. Then, a suitable hash function is applied to the concatenated bit vector to obtain a vector that has a fixed length (usually, 32 or 64 bits) and differs from other generated bit vectors with high probability. Finally, all atom identifiers are updated with the hashed vectors and the entire procedure is repeated until the maximum number of iterations - given by the radius FR - is reached.

Duplicate Structure Removal. All atom identifiers during initialization and all iterations are gathered. In this additional removal step, identifiers referring to the same substructure are removed.

Readout. A fingerprint vector with FS bits is initialized with zeros. Each remaining identifier is converted to an integer i and the bit at position ($i \bmod \mathrm{FS}$) in the fingerprint vector is set to one.

The resulting bit vector is called a Morgan fingerprint. If the hash function is chosen properly and FS is large enough, collisions are unlikely and consequently, different sub-

structures tend to be represented by different bits. While Figure 2 emphasizes the iterative behaviour that the fingerprint algorithm has in common with the Weisfeiler-Lehmann algorithm and graph neural networks, Morgan fingerprints are usually regarded as part of the feature generation. Next, we will shortly describe the baseline methods and how they make use of Morgan fingerprints as input features.

Support Vector Regression (SVR). The support vector regression model uses the support vector machines for function estimation.[14] Two important hyperparameters are the regularization parameter C and the parameter ϵ, which specifies that the loss between the predicted value and the target value vanishes if their distance is smaller than ϵ. As in,[15] we use the RBF kernel and replace the metric term between two points in the Euclidean space by a distance term between two Morgan fingerprints. The resulting function is:

$$\exp(\mathrm{GK}(1 - T(x, x'))^2) \tag{S4}$$

where the kernel coefficient GK is a hyperparameter, x and x' are two bit vectors of size FS and T denotes the Tanimoto similarity index given by

$$T(x, x') = \frac{\sum_{1 \leq i \leq \mathrm{FS}} (x_i \wedge x'_i)}{\sum_{1 \leq i \leq \mathrm{FS}} (x_i \vee x'_i)} \in [0, 1] \tag{S5}$$

Random forests (RF). Random forests (RF) is an ensemble method that can be used for classification and regression. The method constructs a number of decision trees by randomly subsampling from the dataset and by randomly selecting subsets of features.[16] The method makes a prediction by averaging the outcome of the decision trees. Important hyperparameters are the number of trees, the maximum number of samples and the maximum number of features. A full list of the RF hyperparameters and their ranges is provided in Table S2 in the Appendix. In the case of Morgan fingerprints, the fingerprint size corresponds to the number of overall features from which a maximum number of bits is selected for

constructing an individual decision tree.

High Dimensional Model Representation (HDMR). The High Dimensional Model Representation is the final chosen baseline model. The method works by approximating an unknown multivariable function, f, using its finite expansion[17]. In this case, f represents a mapping between a molecular fingerprint vector bit values, x_i and the final power conversion efficiency of an organic heterojunction solar cell, y, with the molecular fingerprint vector generated from the structure of the donor candidate molecule. Under the HDMR representation, the function, f can be approximated using the following expression:

$$y \approx f(x) = f_0 + \sum_{i=1}^{N_x} f_i(x_i) + \sum_{i=1}^{N_x} \sum_{j=1}^{N_x} f_{ij}(x_i, x_j), \tag{S6}$$

where N_x is the dimension of the input space, for this application it is equal to the fingerprint size, FS, and f_0 represents the mean value of $f(x)$. The above approximation is sufficient in many situations in practice since terms containing functions of more than two input parameters can often be ignored due to their negligible contributions compared to the lower-order terms[18]. The terms in Equation S6 are evaluated by approximating the functions $f_i(x_i)$ and $f_{ij}(x_i, x_j)$ with orthonormal basis functions, $\phi_k(x_i)$. In this work, the basis functions have been constructed from ordinary polynomials[18]. The HDMR parameters are listed in Table S2.

S2 Models hyperparameters

Table S1: Neural models hyperparameters.

Parameter name	Parameter value	Parameter sampling
	g-FSI/BiLSTM	
Max sequence length (SEQ)	60, 160	fixed value[a]

Table S1: (Continued)

No. of attention neurons (NA)	SEQ	fixed value
Embedding dimension (ED)	8, 16, 32, 64, 128, 256	categorical
No. of neurons in MLP input layer (NI)	$2 \times$ ED	fixed value
No. of hidden MLP layers (NL)	1 – 4	integer
No. of hidden MLP neurons (HN)	8 – 256	custom-integer[b]
Dropout rate (DR)	$0.0 - 0.3$	uniform
Learning rate (LR)	$10^{-5} - 10^{-2}$	log-uniform
Weight decay rate (WD)	$10^{-5} - 10^{-2}$	log-uniform
	Simple graph neural network	
Embedding dimension (ED)	8, 16, 32, 64, 128, 256	categorical
No. of convolutional layers (CL)	1 – 6	integer
No. of hidden MLP layers (NL)	1 – 4	integer
No. of hidden MLP neurons (HN)	8 – 256	custom-integer[b]
Dropout rate (DR)	$0.0 - 0.3$	uniform
Learning rate (LR)	$10^{-5} - 10^{-2}$	log-uniform
Weight decay rate (WD)	$10^{-5} - 10^{-2}$	log-uniform
	Attentive fingerprint	
Fingerprint size (FS)	8 – 256	integer
No. of atom node layers (AL)	1 – 6	integer
No. of super node layers (SL)	1 – 4	integer
Dropout rate (DR)	$0.0 - 0.3$	uniform
Learning rate (LR)	$10^{-5} - 10^{-2}$	log-uniform
Weight decay rate (WD)	$10^{-5} - 10^{-2}$	log-uniform

Table S1: (Continued)

[a] first value - CEPDB, second value - HOPV15

[b] the number of neurons in the first hidden layer is fixed and equal to the size of the embedding dimension: $\mathrm{HN}_1 = \mathrm{ED}$; the number of neurons in the subsequent layers is sampled from the provided range in decreasing direction (is equal to or smaller than the number of neurons in the previous layer): $\mathrm{HN}_i \leq \mathrm{HN}_{i-1} \quad i = 2, 3, 4$

Table S2: Baseline models hyperparameters.

Parameter name	Parameter value	Parameter sampling
	Fingerprint parameters (all baseline models)	
Fingerprint size (FS)	128, 256, 512, 1024, 2048, 4096	categorical
Fingerprint radius (FR)	2, 3, 4, 5	categorical
Fingerprint chirality (FC)	True	fixed value
Fingerprint bond types (FB)	True	fixed value
	Support vector regression parameters	
SVR kernel (SK)	custom-rbf	-
Gamma kernel coefficient (GK)	10^{-3} – 20.0	loguniform
Regularization parameter (CP)	10^{-1} – 20.0	loguniform
Epsilon parameter (EP)	10^{-4} – 1.0	loguniform
	Random forests parameters	
No. of trees (NT)	16 – 256	integer
Max tree depth (MD)	10 – 100	integer
Min samples split (MSS)	2 – 5	integer
Min samples leaf (MSL)	1 – 5	integer
Max features (MF)	0.05, 0.1, 0.5, 1.0	categorical
Bootstrap (BS)	True, False	categorical
Max samples (MS)	5 – 50	integer
	High dimensional model representation parameters	
Polynomial order (PO)	6	fixed value

Table S2: (Continued)

HDMR order (HO)	2	fixed value

Table S3: Neural models optimal hyperparameters for CEPDB dataset after 100 trials.

Parameter name	Optimal parameter value
g-FSI/BiLSTM	
Max sequence length (SEQ)[a]	60, 160
No. of attention neurons (NA)	SEQ
Embedding dimension (ED)	256
No. of neurons in MLP input layer (NI)	$2 \times$ ED
No. of hidden MLP layers (NL)	1
No. of hidden MLP neurons (HN)	145
Dropout rate (DR)	1.7050×10^{-2}
Learning rate (LR)	1.8521×10^{-3}
Weight decay rate (WD)	1.9480×10^{-5}
Simple graph neural network	
Embedding dimension (ED)	256
No. of convolutional layers (CL)	6
No. of hidden MLP layers (NL)	1
No. of hidden MLP neurons (HN)	144
Dropout rate (DR)	5.9461×10^{-2}
Learning rate (LR)	8.6572×10^{-4}
Weight decay rate (WD)	1.4280×10^{-5}
Attentive fingerprint	
Fingerprint size (FS)	195
No. of atom node layers (AL)	5

Table S3: (Continued)

No. of super node layers (SL)	2
Dropout rate (DR)	1.237×10^{-1}
Learning rate (LR)	5.4619×10^{-4}
Weight decay rate (WD)	1.9332×10^{-5}

[a] first value - CEPDB, second value - HOPV15

Table S4: Baseline models optimal hyperparameters for CEPDB dataset after 100 trials.

Parameter name	Optimal parameter value
Fingerprint parameters (all baseline models)	
Fingerprint size (FS)	4096
Fingerprint radius (FR)	3
Fingerprint chirality (FC)	True
Fingerprint bond types (FB)	True
Support vector regression parameters	
SVR kernel (SK)	custom-rbf
Gamma kernel coefficient (GK)	3.2920×10^{-1}
Regularization parameter (CP)	1.7246×10^{1}
Epsilon parameter (EP)	9.1050×10^{-4}
Random forests parameters	
No. of trees (NT)	180
Max tree depth (MD)	83
Min samples split (MSS)	5
Min samples leaf (MSL)	1
Max features (MF)	0.1
Bootstrap (BS)	False
Max samples (MS)	6
High dimensional model representation parameters	
Polynomial order (PO)	6

Table S4: (Continued)

HDMR order (HO)	2

S3 Random Forests learning curve on HOPV15

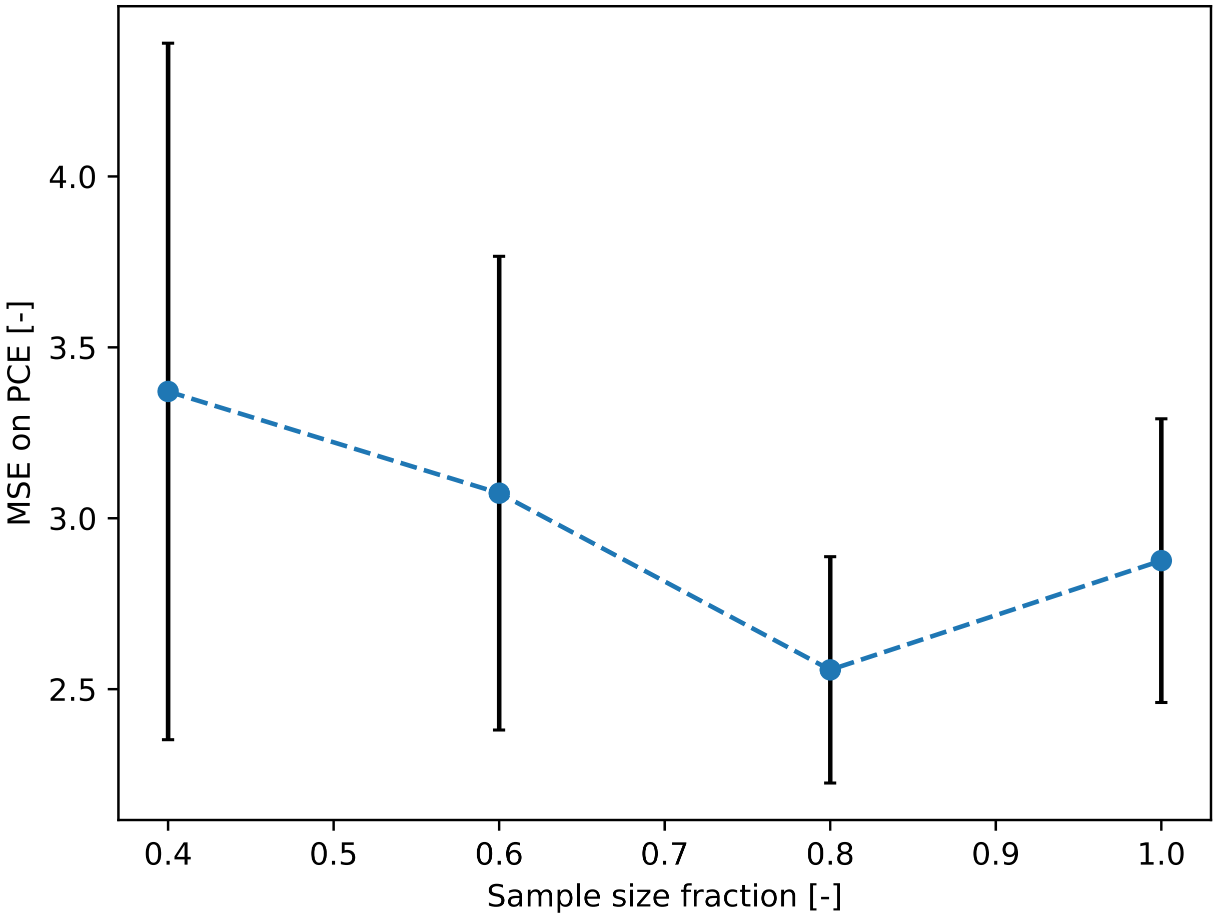

Figure S1: Random Forests learning curve on HOPV15 dataset showing MSE on predicting PCE as a function of the sample size. Error bars are plotted as $\pm\sigma$ calculated from the results on all five outer cross-validation folds.

References

(1) Wu, J.; Wang, S.; Zhou, L.; Ji, X.; Dai, Y.; Dang, Y.; Kraft, M. Deep-Learning Architecture in QSPR Modeling for the Prediction of Energy Conversion Efficiency of Solar Cells. *Industrial & Engineering Chemistry Research* **2020**,

(2) Lambard, G.; Gracheva, E. SMILES-X: autonomous molecular compounds characterization for small datasets without descriptors. *Machine Learning: Science and Technology* **2020**, *1*, 1–11.

(3) Weisfeiler, B.; Lehmann, A. A reduction of a graph to a canonical form and an algebra arising during this reduction. *Nauchno-Technicheskaya Informatsia* **1968**, *2*, 12–16.

(4) Duvenaud, D. K.; Maclaurin, D.; Iparraguirre, J.; Bombarell, R.; Hirzel, T.; Aspuru-Guzik, A.; Adams, R. P. Convolutional networks on graphs for learning molecular fingerprints. *Advances in Neural Information Processing Systems* **2015**, *28*, 2224–2232.

(5) Kipf, T. N.; Welling, M. Semi-supervised classification with graph convolutional networks. *arXiv preprint arXiv:1609.02907* **2016**,

(6) Gilmer, J.; Schoenholz, S. S.; Riley, P. F.; Vinyals, O.; Dahl, G. E. Neural message passing for quantum chemistry. *arXiv preprint arXiv:1704.01212* **2017**,

(7) Xiong, Z.; Wang, D.; Liu, X.; Zhong, F.; Wan, X.; Li, X.; Li, Z.; Luo, X.; Chen, K.; Jiang, H., et al. Pushing the boundaries of molecular representation for drug discovery with the graph attention mechanism. *Journal of Medicinal Chemistry* **2019**,

(8) Wu, Z.; Ramsundar, B.; Feinberg, E. N.; Gomes, J.; Geniesse, C.; Pappu, A. S.; Leswing, K.; Pande, V. MoleculeNet: a benchmark for molecular machine learning. *Chemical Science* **2018**, *9*, 513–530.

(9) Cho, K.; Van Merriënboer, B.; Gulcehre, C.; Bahdanau, D.; Bougares, F.; Schwenk, H.;

Bengio, Y. Learning phrase representations using RNN encoder-decoder for statistical machine translation. *arXiv preprint arXiv:1406.1078* **2014**,

(10) Li, Y.; Tarlow, D.; Brockschmidt, M.; Zemel, R. Gated graph sequence neural networks. *arXiv preprint arXiv:1511.05493* **2015**,

(11) Veličković, P.; Cucurull, G.; Casanova, A.; Romero, A.; Lio, P.; Bengio, Y. Graph attention networks. *arXiv preprint arXiv:1710.10903* **2017**,

(12) Morgan, H. L. The generation of a unique machine description for chemical structures-a technique developed at chemical abstracts service. *Journal of Chemical Documentation* **1965**, *5*, 107–113.

(13) Rogers, D.; Hahn, M. Extended-connectivity fingerprints. *Journal of Chemical Information and Modeling* **2010**, *50*, 742–754.

(14) Smola, A. J.; Schölkopf, B. A tutorial on support vector regression. *Statistics and Computing* **2004**, *14*, 199–222.

(15) Zhao, Z.-W.; del Cueto, M.; Geng, Y.; Troisi, A. Effect of Increasing the Descriptor Set on Machine Learning Prediction of Small Molecule-Based Organic Solar Cells. *Chemistry of Materials* **2020**, *32*, 7777–7787.

(16) Breiman, L. Random forests. *Machine Learning* **2001**, *45*, 5–32.

(17) Rabitz, H.; Aliş, Ö. F. General foundations of high-dimensional model representations. *Journal of Mathematical Chemistry* **1999**, *25*, 197–233.

(18) Li, G.; Wang, S.-W.; Rabitz, H. Practical approaches to construct RS-HDMR component functions. *The Journal of Physical Chemistry A* **2002**, *106*, 8721–8733.
